# Supplementary material for: Design, Synthesis and Biological Evaluation of Novel Osimertinib-Based HDAC and EGFR Dual Inhibitors
Source: Molecules. 2019 Jun 29;24(13):2407. doi: 10.3390/molecules24132407 (PMC6651501; doi:10.3390/molecules24132407)
Supplement: Supplementary file 1 [file molecules-24-02407-s001.pdf]

Supporting information

Design, Synthesis and Biological Evaluation of Novel Osimertinib-based HDAC and EGFR Dual Inhibitors

Hang Dong, Hao Yin, Chunlong Zhao, Jiangying Cao, Wenfang Xu, Yingjie Zhang*

Representative ^{1}H and ^{13}C NMR spectra

Contents

1. ^{1}H NMR and ^{13}C NMR spectral information of **5B** 2
2. ^{1}H NMR and ^{13}C NMR spectral information of **5E** 3
3. ^{1}H NMR and ^{13}C NMR spectral information of **10A** 4
4. ^{1}H NMR and ^{13}C NMR spectral information of **9C** 5
5. ^{1}H NMR and ^{13}C NMR spectral information of **9E** 6

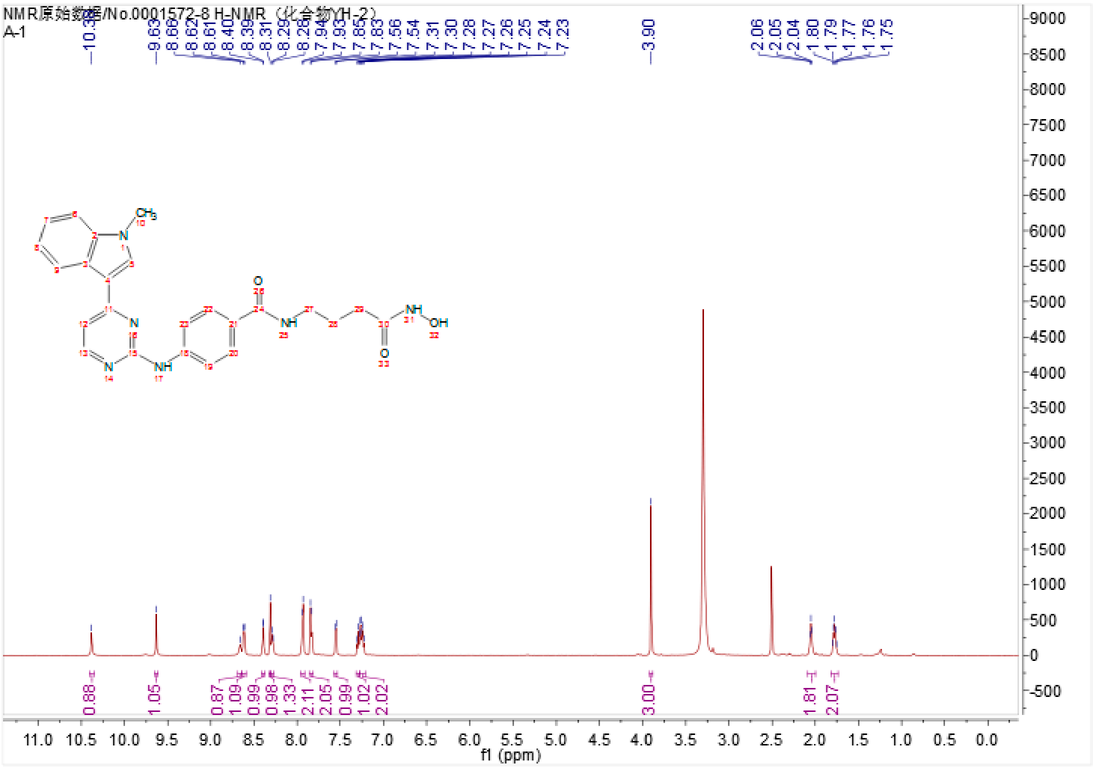

^{1}H NMR of **5B**

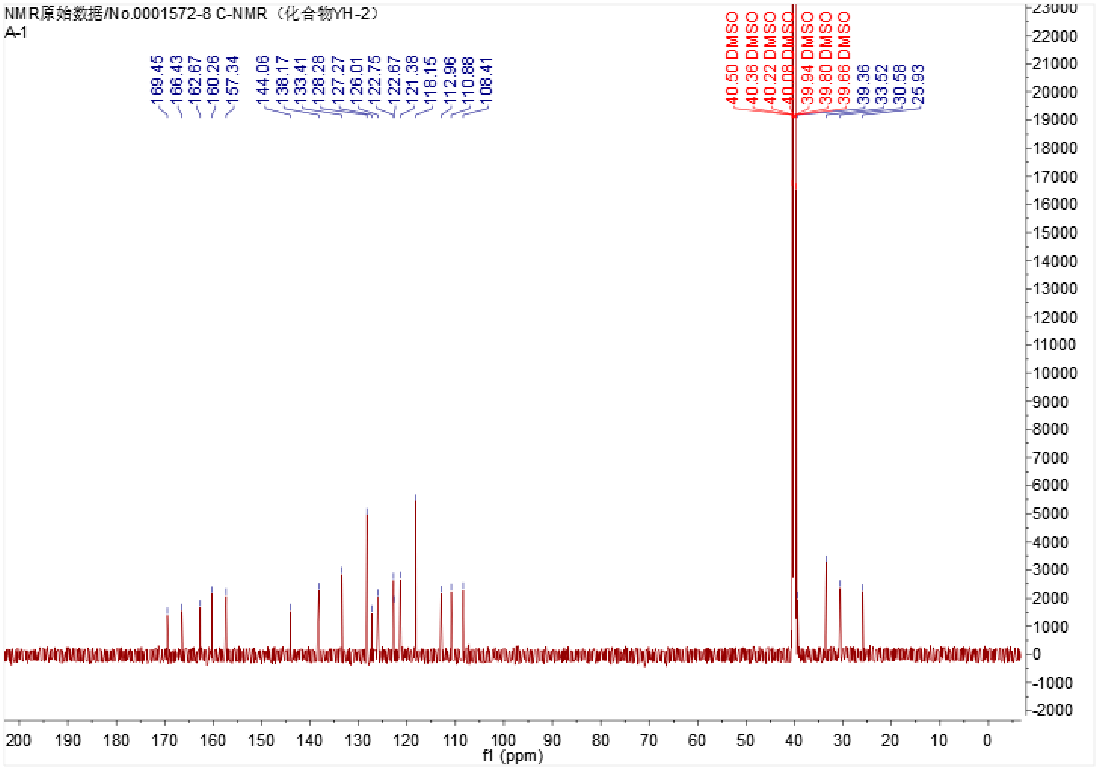

^{13}C NMR of **5B**

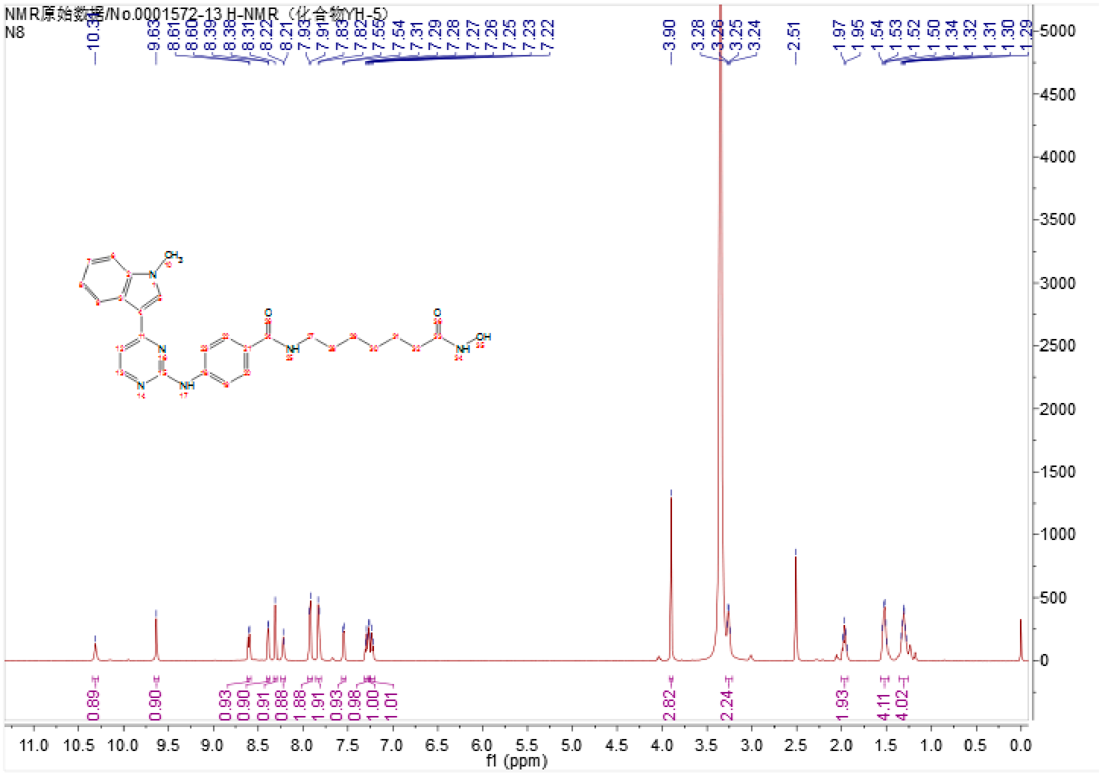

^{1}H NMR of **5E**

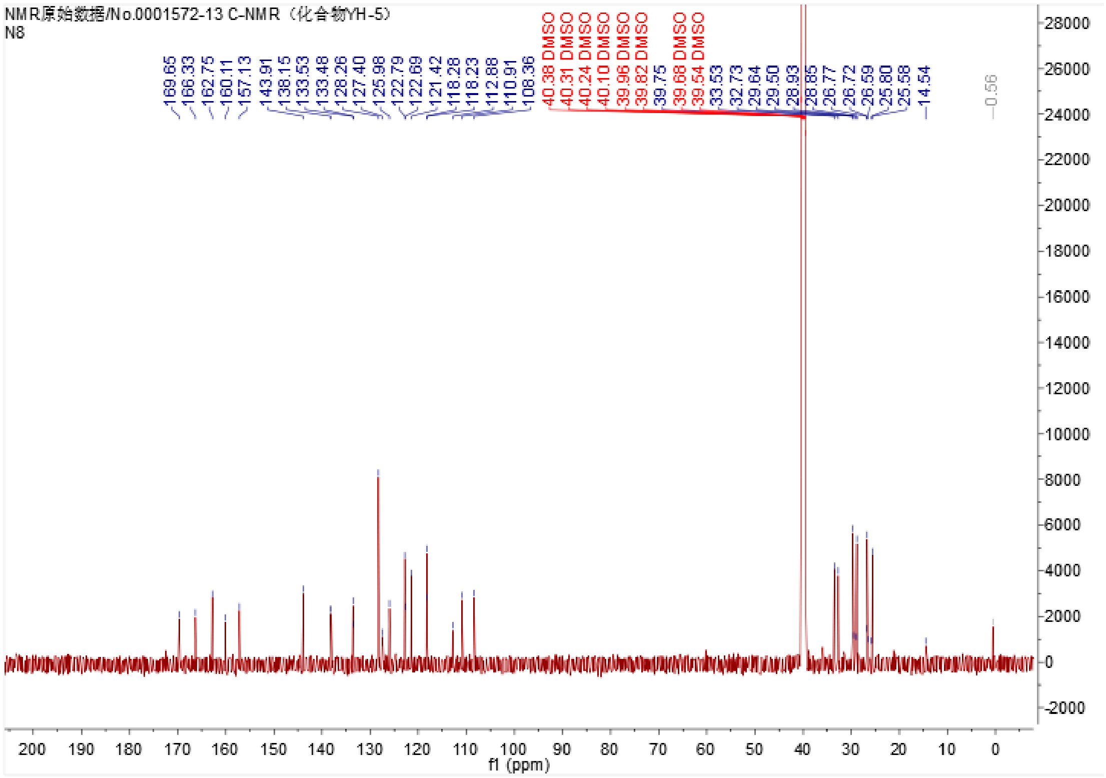

^{13}C NMR of **5E**

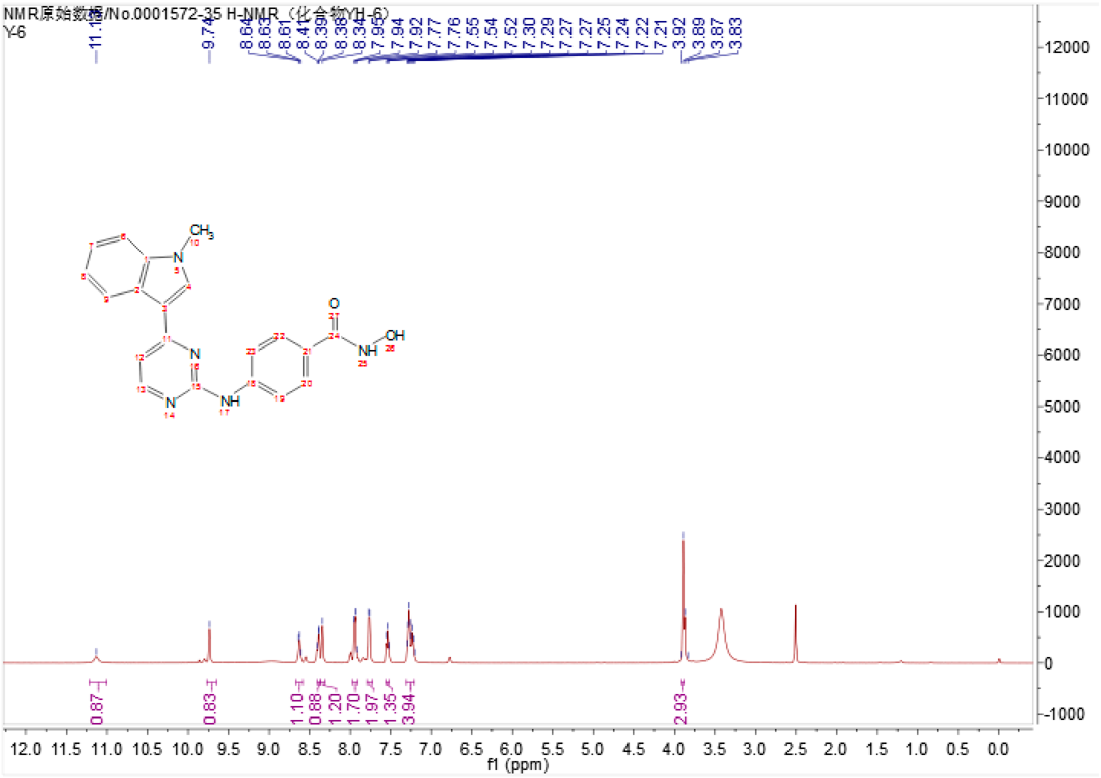

^1H NMR of **10A**

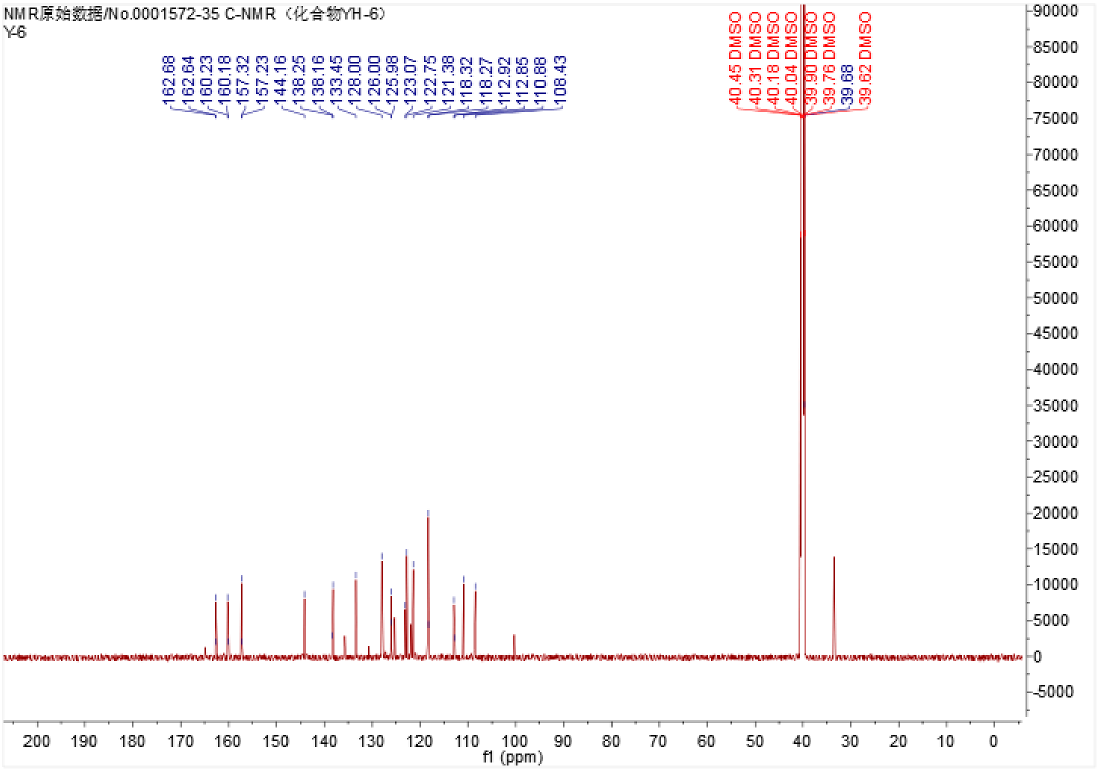

^{13}C NMR of **10A**

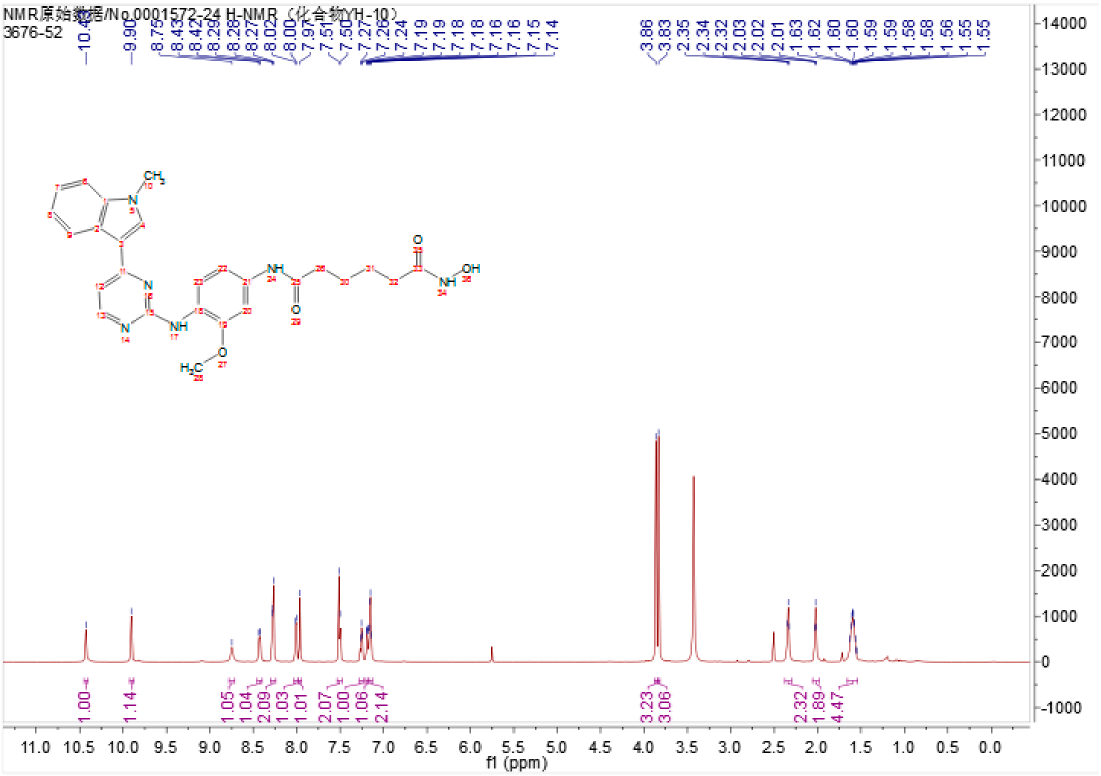

^{1}H NMR of **9C**

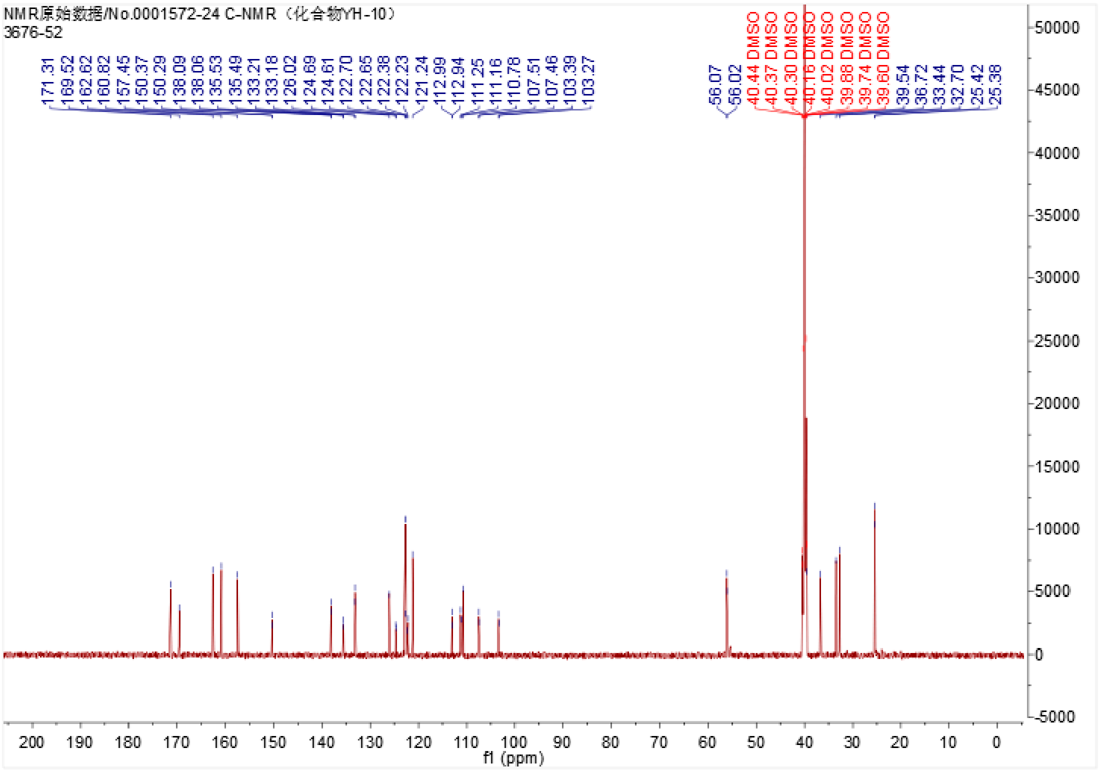

^{13}C NMR of **9C**

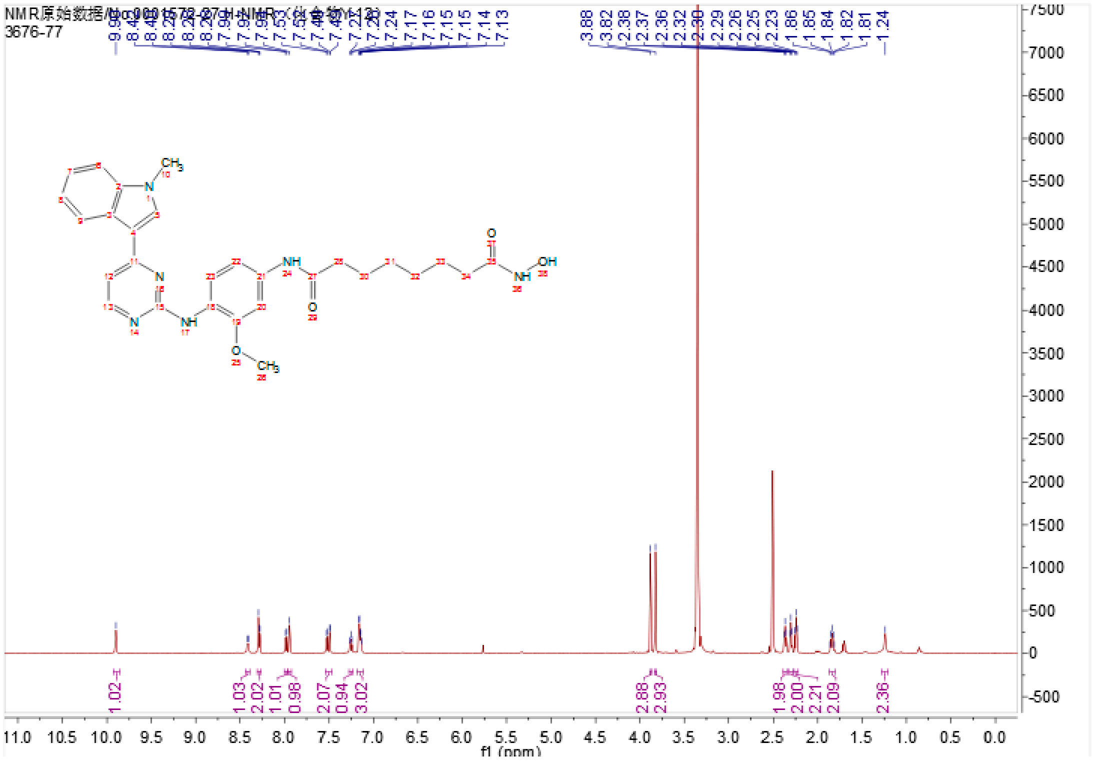

^{1}H NMR of **9E**

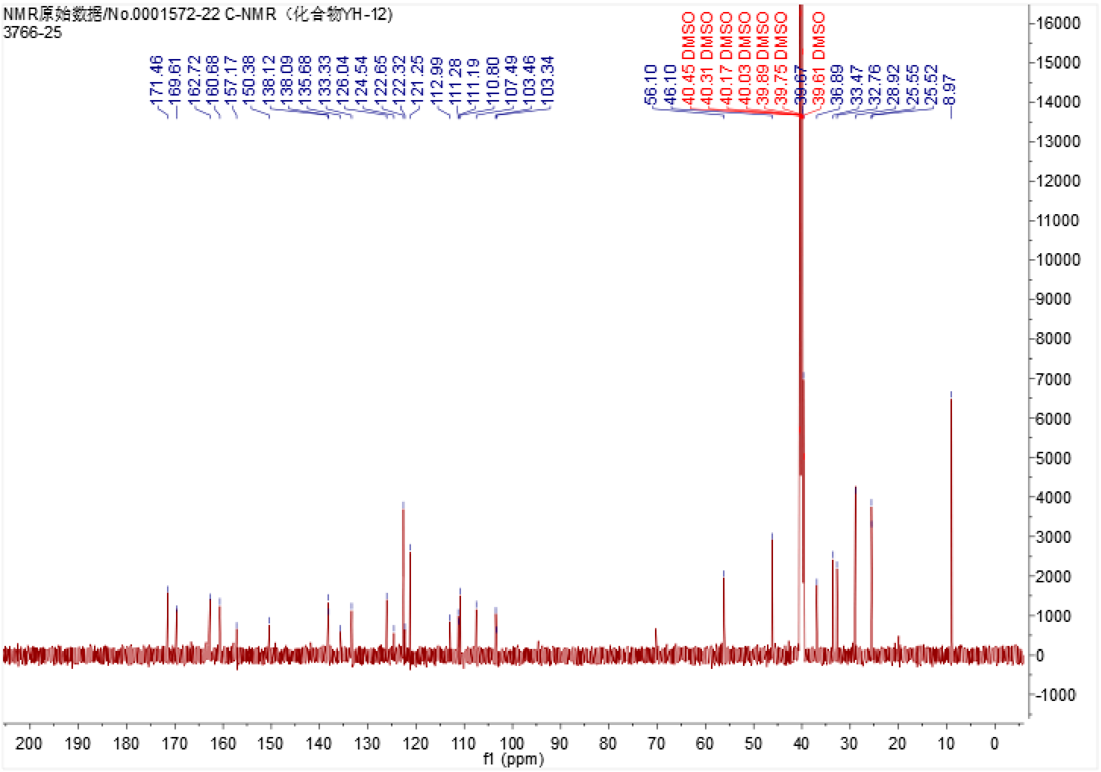

^{13}C NMR of **9E**
